# INDIVIDUAL AND INTERPERSONAL INFLUENCERS OF MEALTIME EXPERIENCES: PERSPECTIVES OF NURSING ASSISTANTS

**DOI:** 10.1093/geroni/igz038.1867

**Published:** 2019-11-08

**Authors:** Joy W Douglas, Seung Eun Jung, Hyunjin Noh, Amy Ellis, Christine Ferguson

**Affiliations:** 1 University of Alabama, Tuscaloosa, Alabama, United States; 2 The University of Alabama School of Social Work, Tuscaloosa, Alabama, United States

## Abstract

In the United States, long-term care Certified Nursing Assistants (CNAs)’s central role is to provide direct care to residents, including mealtime assistance. It has been reported that employee turnover among CNAs is nearly 75% annually. High turnover rates of CNAs can increase the workload for remaining CNAs, interrupt quality of care for residents, and require extra resources for recruiting new staff. The aim of this qualitative study was to explore the individual and interpersonal barriers and facilitators CNAs experience when providing mealtime assistance to residents with dementia. Using purposive sampling, nine focus groups were conducted with a total of 53 CNAs who had at least one year of experience as a CNA working with older adults. Focus group questions were developed using the Social Ecological Model. All focus groups were audio recorded and transcribed verbatim. Data were analyzed using the directed content analysis approach. At the individual level, CNAs identified that communication skills with residents and coworkers, and the ability to accurately interpret resident behavior positively affected their ability to provide mealtime assistance. At the interpersonal level, interdisciplinary collaboration was identified as a significant facilitator. Reported barriers included negative interference from residents’ family members, unpredictable resident behaviors, and lack of support from coworkers. CNAs reported individual and interpersonal factors that may influence their ability to effectively feed residents with dementia. Our findings will inform future investigations regarding job turnover. Equally important, providing CNAs with the training and opportunity to perform their duties efficiently can ultimately benefit the residents’ mealtime experiences.

